# Supplementary material for: Effectiveness of Social Inclusion Interventions for Anxiety and Depression among Adolescents: A Systematic Review
Source: Int J Environ Res Public Health. 2023 Jan 19;20(3):1895. doi: 10.3390/ijerph20031895 (PMC9914997; doi:10.3390/ijerph20031895)
Supplement: Supplementary file 1 [file ijerph-20-01895-s001.zip › Supporting information_PROSPERO protocol (S3)]

Systematic review

A list of fields that can be edited in an update can be found here

1. * Review title.

Give the title of the review in English

Effectiveness of social inclusion interventions for improving anxiety and depression outcomes among adolescents: a participatory systematic review

2. Original language title.

For reviews in languages other than English, give the title in the original language. This will be displayed with the English language title.

3. * Anticipated or actual start date.

Give the date the systematic review started or is expected to start.

03/08/2021

4. * Anticipated completion date.

Give the date by which the review is expected to be completed.

03/08/2022

5. * Stage of review at time of this submission.

This field uses answers to initial screening questions. It cannot be edited until after registration.

Tick the boxes to show which review tasks have been started and which have been completed.

Update this field each time any amendments are made to a published record.

The review has not yet started: Yes

Review stage	Started	Completed
Preliminary searches	No	No
Piloting of the study selection process	No	No
Formal screening of search results against eligibility criteria	No	No
Data extraction	No	No
Risk of bias (quality) assessment	No	No
Data analysis	No	No

Provide any other relevant information about the stage of the review here.

6. * Named contact.

The named contact is the guarantor for the accuracy of the information in the register record. This may be any member of the review team.

Xanthe Hunt

Email salutation (e.g. "Dr Smith" or "Joanne") for correspondence:

Dr Hunt

7. * Named contact email.

Give the electronic email address of the named contact.

xanthe@sun.ac.za

8. Named contact address

Give the full institutional/organisational postal address for the named contact.

Room 4009, Education Building, Faculty of Medicine and Health Sciences, Stellenbosch Unviersity

9. Named contact phone number.

Give the telephone number for the named contact, including international dialling code.

0820840939

10. * Organisational affiliation of the review.

Full title of the organisational affiliations for this review and website address if available. This field may be completed as 'None' if the review is not affiliated to any organisation.

Stellenbosch University

Organisation web address:

http://www.sun.ac.za/english

11. * Review team members and their organisational affiliations.

Give the personal details and the organisational affiliations of each member of the review team. Affiliation refers to groups or organisations to which review team members belong. **NOTE: email and country now MUST be entered for each person, unless you are amending a published record.**

Dr Xanthe Hunt. Institute for Life Course Health Research, Department of Global Health, Faculty of Medicine and Health Sciences, Stellenbosch University
Professor Jason Bantjes. Institute for Life Course Health Research, Department of Global Health, Faculty of Medicine and Health Sciences, Stellenbosch University
Professor Tom Shakespeare. London School of Hygiene and Tropical Medicine
Gabriele Vilyte. Stellenbosch University
Mr Selvan Naidoo. Institute for Life Course Health Research, Department of Global Health, Faculty of Medicine and Health Sciences, Stellenbosch University
Ms Rachel Mbuyamba. Institute for Life Course Health Research, Department of Global Health, Faculty of Medicine and Health Sciences, Stellenbosch University

12. * Funding sources/sponsors.

Details of the individuals, organizations, groups, companies or other legal entities who have funded or sponsored the review.

This systematic review is supported by the Wellcome Trust's Commission on Active Ingredients for Youth Anxiety and Depression.

Grant number(s)

State the funder, grant or award number and the date of award

13. * Conflicts of interest.

List actual or perceived conflicts of interest (financial or academic).

None

14. Collaborators.

Give the name and affiliation of any individuals or organisations who are working on the review but who are not listed as review team members. **NOTE: email and country must be completed for each person, unless you are amending a published record.**

15. * Review question.

State the review question(s) clearly and precisely. It may be appropriate to break very broad questions down into a series of related more specific questions. Questions may be framed or refined using PI(E)COS or similar where relevant.

1. What types of social inclusion interventions are being delivered to adolescents with the goal of preventing and treating anxiety and depression among adolescents?

2. At what time points are these being delivered (e.g., universal population level interventions focussed on prevention; selective interventions (focussed on at-risk populations) and indicated interventions which promote recovery)?

3. How effective are social inclusion interventions at improving mental health and preventing mental health conditions among adolescents?

4. Are there specific groups of adolescents (e.g., LGBTQ+, disabled) for whom social inclusion interventions are most effective?

16. * Searches.

State the sources that will be searched (e.g. Medline). Give the search dates, and any restrictions (e.g. language or publication date). Do NOT enter the full search strategy (it may be provided as a link or attachment below.)

Electronic Searches:

• MEDLINE(R)

• Embase Classic+Embase

• PsycINFO

• CAB Global Health

• CINAHL

• ERIC

• CENTRAL

• Scopus

• Web of Science (Social Sciences Citation Index)

• WHO Global Health Index

Searching other resources:

We will search the reference lists of identified recent papers and reviews.

We will also search the following databases:

1. ClinicalTrials.gov
2. CurrentControlledTrials.com
3. Open Access Theses and Dissertations
4. ProQuest Dissertations and Theses Global
5. OpenGrey
6. Digital Access to Research Theses (DART)?
7. Europe E?theses Portal

We will also conduct hand-searches of specific journals which are likely to have much relevant content, including the journal Social Inclusion and Mental Health.

Language:

Only English language studies will be eligible for inclusion.

Time:

There will be no time restrictions applied in the search for relevant studies.

17. URL to search strategy.

Upload a file with your search strategy, or an example of a search strategy for a specific database, (including the keywords) in pdf or word format. In doing so you are consenting to the file being made publicly accessible. Or provide a URL or link to the strategy. Do NOT provide links to your search **results**.

Alternatively, upload your search strategy to CRD in pdf format. Please note that by doing so you are consenting to the file being made publicly accessible.

Do not make this file publicly available until the review is complete

18. * Condition or domain being studied.

Give a short description of the disease, condition or healthcare domain being studied in your systematic review.

Adolescence signifies numerous physical, cognitive, social and emotional changes in a person’s life (Sawyer et al., 2012). These developmental changes coupled with adolescents’ social, cultural and economic environments can render them vulnerable to mental health issues (Balvin & Banati, 2017). Depression and anxiety remain some of the leading causes of disability among the world’s adolescents (WHO, 2020). At their worst, if left untreated, they can lead to suicide, which is the third leading cause of death among older adolescents (15-19 years) (WHO, 2020).

Mental health issues, get further compounded for adolescents coming from marginalised groups, such as those living in poverty or with disability, racial minorities, LGBTQ+ and refugee youth (Sapiro & Ward, 2020). According to the WHO (2020) “nearly 90% of the world’s adolescents live in low-or middle-income countries and more than 90% of adolescent suicides are among adolescents living in those countries”.

Social withdrawal associated with mental illness can exacerbate isolation and loneliness, thus further worsening symptoms (WHO, 2020).

19. * Participants/population.

Specify the participants or populations being studied in the review. The preferred format includes details of both inclusion and exclusion criteria.

Studies involving adolescents where at least 50% of the total sample are adolescents (aged 14 to under the age of 24 years old) will be included or where age-disaggregated data can be extracted from samples which are mixed in terms of age. There will be no restrictions as to gender, ethnicity, country, severity of depression and anxiety symptoms, or comorbidities.

Studies with participants who are younger than 14 years, and/or older than 24 years will be excluded. If data combines adolescent and adult populations, and if the effects for the adolescent population cannot be separately estimated, that sample will be excluded.

20. * Intervention(s), exposure(s).

Give full and clear descriptions or definitions of the interventions or the exposures to be reviewed. The preferred format includes details of both inclusion and exclusion criteria.

Any form of social inclusion interventions, provided alone or in addition to treatment as usual, delivered to the target population will be included, such as:

1. Community-based (i.e. eco-systemic) interventions and outreach, psychological and counselling support, media campaigns, career development initiatives and educational programs.

2. All SI interventions aimed at dismantling the barriers which prevent people from fully participating in society

3. Universal population level interventions focussed on prevention.

4. Selective interventions (focussed on at-risk populations).

5. Indicated interventions which promote recovery.

6. Interventions in specific groups of adolescents such as marginalised adolescents, those with disabilities, racial minorities, LGBTQ+, and refugee youth.

7. Social inclusion interventions that go beyond programmes targeting the social determinants of adolescent wellbeing by accounting for the systemic and structural factors,

8. Interventions that include social skills, vocational, or educational classroom-based training, counselling or one-to-one support, internships, placements, on-the-job or occupational training, financial incentives, case management, and individual support.

9. Studies where the purpose of the intervention is to improve community inclusion, social participation, sense of belonging, access to learning, cultural and social opportunities or social relationships in the community.

10. Mental health interventions effecting change in school, community, and broader socio-political environments.

11. Studies of multi-component interventions

21. * Comparator(s)/control.

Where relevant, give details of the alternatives against which the intervention/exposure will be compared (e.g. another intervention or a non-exposed control group). The preferred format includes details of both inclusion and exclusion criteria.

Relevant comparison groups include (i) adolescents not exposed to the intervention being investigated, (ii) adolescents exposed to other forms of interventions included as treatment as usual, (iii) adolescents exposed to other interventions for social inclusion, (iv) adolescents not exposed to any intervention or exposed to placebo/sham therapy.

22. * Types of study to be included.

Give details of the study designs (e.g. RCT) that are eligible for inclusion in the review. The preferred format includes both inclusion and exclusion criteria. If there are no restrictions on the types of study, this should be stated.

Eligible quantitative study designs will include those in which one of the following is true:

-participants are randomly assigned,

-quasi-random assignment is used,

-participants are non-randomly assigned but matched on pre-tests and/or relevant demographic characteristics (e.g., observables/propensity scores) and/or according to a cut-off on an ordinal or continuous variable (regression discontinuity design),

-participants are non-randomly assigned, but statistical methods have been used to control for differences between groups (e.g., multiple regression analysis/instrumental variables regression),

-the design attempts to detect whether the intervention has had an effect significantly greater than any underlying trend over time, using observations at multiple time points before and after the intervention (interrupted time-series design),

-participants receiving an intervention are compared with a similar group from the past who did not (i.e., a historically controlled study),

-observations are made on a group of individuals before and after an intervention, but with no control group (single-group before-and-after study).

23. Context.

Give summary details of the setting or other relevant characteristics, which help define the inclusion or exclusion criteria.

All contexts will be considered.

24. * Main outcome(s).

Give the pre-specified main (most important) outcomes of the review, including details of how the outcome is defined and measured and when these measurement are made, if these are part of the review inclusion criteria.

• Depressive symptoms: measured by self-report measures, clinician-report measures, or recorded by study investigator

• Anxiety symptoms: measured by self-report measures, clinician-report measures, or recorded by study investigator

• Adverse effects (e.g. suicide)

Measures of effect

Please specify the effect measure(s) for you main outcome(s) e.g. relative risks, odds ratios, risk difference, and/or 'number needed to treat.

If possible, we will collect effect sizes and conduct effect size calculations where none are published.

We will convert these effect sizes to a common metric and will present these in forest plots.

- For continuous outcomes, effects sizes with 95% confidence intervals will be calculated, where means and standard deviations are available. If means and standard deviations are not available, we will calculate

standardised mean differences (SMDs) from F ratios, t values, ?2 values and correlation coefficients, where available, using the methods suggested by Lipsey & Wilson (2001).

- For dichotomous outcomes, we will calculate odds ratios with 95% confidence intervals. employment outcomes (e.g., presence or absence of gaining competitive employment), are examples of relevant dichotomous outcomes in this review.

There are statistical approaches available to re?express dichotomous and continuous data to be pooled together (Sánchez?Meca et al., 2003). In order to calculate common metric odds ratios will be converted to SMD effect sizes using the Cox transformation. We will only transform dichotomous effect sizes to SMD if appropriate.

25. * Additional outcome(s).

List the pre-specified additional outcomes of the review, with a similar level of detail to that required for main outcomes. Where there are no additional outcomes please state ‘None’ or ‘Not applicable’ as appropriate to the review

• Self-Efficacy
• Well-Being

• Comorbid mental illness symptoms

• Quality of Life

• Psychosocial and Global Functioning (e.g. increased number of friendships; academic improvement)

Measures of effect

Please specify the effect measure(s) for you additional outcome(s) e.g. relative risks, odds ratios, risk difference, and/or 'number needed to treat.

If possible, we will collect effect sizes and conduct effect size calculations where none are published.

We will convert these effect sizes to a common metric and will present these in forest plots.

- For continuous outcomes, effects sizes with 95% confidence intervals will be calculated, where means and standard deviations are available. If means and standard deviations are not available, we will calculate standardised mean differences (SMDs) from F ratios, t values, ?2 values and correlation coefficients, where available, using the methods suggested by Lipsey & Wilson (2001).

- For dichotomous outcomes, we will calculate odds ratios with 95% confidence intervals. employment outcomes (e.g., presence or absence of gaining competitive employment), are examples of relevant dichotomous outcomes in this review.

There are statistical approaches available to re?express dichotomous and continuous data to be pooled

together (Sánchez?Meca et al., 2003). In order to calculate common metric odds ratios will be converted to SMD effect sizes using the Cox transformation. We will only transform dichotomous effect sizes to SMD if appropriate.

26. * Data extraction (selection and coding).

Describe how studies will be selected for inclusion. State what data will be extracted or obtained. State how this will be done and recorded.

Study Selection:

We will use Rayyan (https://www.rayyan.ai/) to help assess the search results. Unique references will first be screened for relevance by title and abstract by two independent reviewers with disagreement resolved by discussion or third reviewer. The full text of potentially relevant articles will then also be screened independently by two independent reviewers with disagreement resolved by discussion or third reviewer for inclusion. Any discrepancy will be resolved by consensus and discussion with the senior author (XH).

Data Extraction:

Two review authors will independently code and extract data from included studies. A coding sheet will be piloted on several studies and revised as necessary. Disagreements will be resolved by discussion or by consulting a third review author with extensive content and methods expertise. Where an agreement cannot be reached, this will be reported. Data and information will be extracted on: study region (country and world bank classification of the country), available characteristics of participants, intervention characteristics and control conditions, randomisation procedures, research design, sample size, risk of bias and outcomes, and results., follow up period, drop-out rates, assessment (outcome) measures / instruments used, assessment of any process measures. Extracted data will be stored electronically in Microsoft Excel. Studies will be coded by intervention, outcomes and a range of filters such as study design and location. In case of missing information, the author(s) of the original study will be contacted. We will document correspondence with study authors.

27. * Risk of bias (quality) assessment.

State which characteristics of the studies will be assessed and/or any formal risk of bias/quality assessment tools that will be used.

We will use a tool containing seven criteria:

1.Study design (Potential confounders taken into account): well-designed control group, preferably based on random assignment, or an estimation technique which controls for confounding and the associated possibility of selection bias.

2.Masking (RCTs only, also known as blinding)

3.Presence of a power calculation: many studies may be underpowered, but it is difficult to assess without

the inclusion in the study of a power calculation.

4.Attrition: we will apply the US Institute of Education Sciences What Works Clearing House standards for acceptable levels of attrition.

5.Inclusion criteria and measure/s are clearly defined and reliable

6.Clear definition of outcome measures: studies should clearly state the outcomes being used with a definition and the basis on which they are measured, preferably with reference to a widely-used international standard.

7.Baseline balance between treatment and comparison groups.

Confidence in study findings will be rated high, medium or low, for each of the criteria. Overall study quality will be the lowest rating achieved across the criteria – the weakest link in the chain principle. Two review authors will independently apply the risk of bias tool. Disagreements will be resolved by discussion or by consulting a third review author.

28. * Strategy for data synthesis.

Describe the methods you plan to use to synthesise data. This **must not be generic text** but should be **specific to your review** and describe how the proposed approach will be applied to your data. If meta-analysis is planned, describe the models to be used, methods to explore statistical heterogeneity, and software package to be used.

Meta-analysis will only be used if treatments, participants and the underlying clinical question are similar enough for pooling. Otherwise, a narrative description will be provided. Given the potential heterogeneity of social inclusion intervention approaches, a random?effects model will be used in all analyses.

For each sub-outcome, a narrative summary will be prepared for the main themes and findings, including consideration of where there is strong evidence for effect, where there are evidence gaps, and the quality of the evidence. We will conduct a meta-analysis of results by sub-group if there are sufficient number of studies (n=4, (Fu et al., 2011) and our conditions for a meta-analysis are met.

Subgroup analysis and investigation of heterogeneity

We will examine heterogeneity both in the subject matter of included studies (context, intervention and outcomes) and in the reported effect sizes (visually and using I^2). If meta-analysis is appropriate, we will calculate an inverse variance weighted average effect size using a random effects model. However, if there is too much heterogeneity in the reporting of quantitative data, and the effect sizes, we will synthesise the data only narratively, and without a meta-analysis. Heterogeneity will be assessed by comparing study characteristics such as type of intervention and control comparators, participant demographics, quality of trials (randomisation, blinding, losses to follow?up) and outcomes measured. Statistical heterogeneity will be

assessed visually and by examining the I² statistic. This will be supplemented by the ?² test, where a P value 0.05 indicates heterogeneity of intervention effects. In addition, we will estimate and present ?², along with its CIs, as an estimate of the magnitude of variation between studies. This will provide an estimate of the amount of between?study variation. Sensitivity and subgroup analyses will also be used to investigate possible sources of heterogeneity.

29. * Analysis of subgroups or subsets.

State any planned investigation of ‘subgroups’. Be clear and specific about which type of study or participant will be included in each group or covariate investigated. State the planned analytic approach.

If possible, we would like to perform subgroup analyses based on the following characteristics:

1. Type of minority group (LGBTQ+, disabled, refugees, mentally ill, racial minority)

2. Age group (14-19 and 19-24)

3. Type of social inclusion intervention

4. Modality of treatment ? individual versus group intervention.

30. * Type and method of review.

Select the type of review, review method and health area from the lists below.

Type of review

Cost effectiveness
No

Diagnostic
No

Epidemiologic
No

Individual patient data (IPD) meta-analysis
No

Intervention
Yes

Living systematic review
No

Meta-analysis
Yes

Methodology
No

Narrative synthesis
Yes

Network meta-analysis
No

Pre-clinical
No

Prevention
Yes

Prognostic
No

Prospective meta-analysis (PMA)
No

Review of reviews
No

Service delivery
No

Synthesis of qualitative studies
No

Systematic review
Yes

Other
No

Health area of the review

Alcohol/substance misuse/abuse
No

Blood and immune system
No

Cancer
No

Cardiovascular
No

Care of the elderly
No

Child health
No

Complementary therapies
No

COVID-19
No

Crime and justice
No

Dental
No

Digestive system
No

Ear, nose and throat
No

Education
No

Endocrine and metabolic disorders

No

Eye disorders
No

General interest
No

Genetics
No

Health inequalities/health equity
No

Infections and infestations
No

International development
No

Mental health and behavioural conditions
Yes

Musculoskeletal
No

Neurological
No

Nursing
No

Obstetrics and gynaecology
No

Oral health
No

Palliative care
No

Perioperative care
No

Physiotherapy
No

Pregnancy and childbirth
No

Public health (including social determinants of health)
Yes

Rehabilitation
No

Respiratory disorders
No

Service delivery
No

Skin disorders
No

Social care
No

Surgery
No

Tropical Medicine
No

Urological
No

Wounds, injuries and accidents
No

Violence and abuse
No

31. Language.

Select each language individually to add it to the list below, use the bin icon to remove any added in error.
English

There is an English language summary.

32. * Country.

Select the country in which the review is being carried out. For multi-national collaborations select all the countries involved.

England
South Africa

33. Other registration details.

Name any other organisation where the systematic review title or protocol is registered (e.g. Campbell, or The Joanna Briggs Institute) together with any unique identification number assigned by them. If extracted data will be stored and made available through a repository such as the Systematic Review Data Repository (SRDR), details and a link should be included here. If none, leave blank.

34. Reference and/or URL for published protocol.

If the protocol for this review is published provide details (authors, title and journal details, preferably in Vancouver format)

Add web link to the published protocol.

Or, upload your published protocol here in pdf format. Note that the upload will be publicly accessible.

No I do not make this file publicly available until the review is complete

Please note that the information required in the PROSPERO registration form must be completed in full even if access to a protocol is given.

35. Dissemination plans.

Do you intend to publish the review on completion?

Yes

Give brief details of plans for communicating review findings.?

In addition to producing a report for the funders of this review, which will be made available free of charge on their website, a paper will be submitted to a leading journal in this field. A summary report will be prepared and sent to lead clinicians and healthcare professionals within the area of adolescent mental health.

36. Keywords.

Give words or phrases that best describe the review. Separate keywords with a semicolon or new line. Keywords help PROSPERO users find your review (keywords do not appear in the public record but are included in searches). Be as specific and precise as possible. Avoid acronyms and abbreviations unless these are in wide use.

systematic review; participatory systematic review; social inclusion; social exclusion; social inclusion interventions; adolescent mental health; youth mental health; adolescent depression; youth depression; adolescent anxiety; youth anxiety

37. Details of any existing review of the same topic by the same authors.

If you are registering an update of an existing review give details of the earlier versions and include a full bibliographic reference, if available.

38. * Current review status.

Update review status when the review is completed and when it is published.New registrations must be ongoing so this field is not editable for initial submission.
Please provide anticipated publication date

Review_Ongoing

39. Any additional information.

Provide any other information relevant to the registration of this review.

40. Details of final report/publication(s) or preprints if available.

Leave empty until publication details are available OR you have a link to a preprint (NOTE: this field is not editable for initial submission). List authors, title and journal details preferably in Vancouver format.

Give the link to the published review or preprint.
