# Supplementary material for: Effectiveness of Social Inclusion Interventions for Anxiety and Depression among Adolescents: A Systematic Review
Source: Int J Environ Res Public Health. 2023 Jan 19;20(3):1895. doi: 10.3390/ijerph20031895 (PMC9914997; doi:10.3390/ijerph20031895)
Supplement: Supplementary file 1 [file ijerph-20-01895-s001.zip › Supporting information_Quality tool (S2).pdf]

Hunt et al. Supplementary Materials

1. Full search strategy

The search strategy was refined for each database but included the following terms:

Population. Adolescen* OR Child* OR Teen* OR Youth* OR Student* OR Pupil OR Learner OR Pube* AND

Intervention. Social inclusion OR social exclusion OR Social* exclu* OR social* inclu* OR acceptance OR sense of belonging OR anti-bullying OR school climate OR social acceptance OR social capital OR discrimination OR exclusion OR isolation OR participation OR social justice OR recreation OR Participat* OR integrat* OR Social network OR cohesion OR lonel* AND

Outcome. Anx* OR depress*

2. Quality appraisal tool standards

1. Study design (Potential confounders taken into account): impact evaluations need either a well-designed control group, preferably based on random assignment, or an estimation technique which controls for confounding and the associated possibility of selection bias.
2. Masking (RCTs only, also known as blinding): masking helps limit the biases which can occur if study participants, data collectors or data analysts are aware of the assignment condition of individual participants.
3. Attrition can be a major source of bias in studies, especially if these is differential attrition between the treatment and comparison group so that the two may no longer be balanced in pre-intervention characteristics. The US Institute of Education Sciences What Works Clearing House has developed standards for acceptable levels of attrition, in aggregate and the differential, which we will apply.[1]
4. Inclusion criteria and measure/s is clearly defined and reliable: for a study to be useful the study population must be clear, preferably with reference to a widely-used international standard
5. Clear definition of outcome measures is needed in order to aid interpretation and reliability of findings and comparability with other studies. Studies should clearly state the outcomes being used with a definition and the basis on which they are measured, preferably with reference to a widely-used international standard.
6. Baseline balance shows that the treatment and comparison groups are the same at baseline. Lack of balance can bias the results.

[1] See table 1 https://homvee.acf.hhs.gov/HomVEE-Attrition-White_Paper-7-2015.pdf.

Appendix: Study quality assessment criteria

	Criterion	Low	Medium	High
1	Study design (Potential confounders taken into account)	Before versus after. Naïve matching	IV, RDD, PSM, double difference	RCT, natural experiment
2	Blinding (RCTs only)	No mention of blinding	Blinding for analysis.	Blinding of data collection (where feasible). Blinding for analysis.
3	Losses to follow up are presented and acceptable	Attrition not reported, OR falls well outside WWC acceptable combined levels*	Overall and differential attrition close to WWC combined levels*	Overall and differential attrition within WWC combined levels*
4	Inclusion criteria and measure/s is clearly defined and reliable	No definition OR overall attrition > 50%	Unclear definition OR Single question item only	Clear definition
5	Outcome measures are clearly defined and reliable	No definition	Unclear definition	Clear definition using existing measure where possible
6	Baseline balance (N.A. for before versus after)	No baseline balance test (except RCT) OR reported and significant differences on more than five measures. PSM without establishing common support.	Baseline balance test, imbalance on 5 or fewer measures	RCT, RDD
	Overall confidence in study findings	Low on any item	Medium or high confidence on all items	RCT with high confidence on all items
